# High Rate of Obstetric Complications in Patients With Essential Thrombocythemia

**DOI:** 10.7759/cureus.20449

**Published:** 2021-12-15

**Authors:** Dicle İskender, Seval Yılmaz-Ergani, Munevver Aksoy, Betul Tokgoz, Mujde Can Ibanoglu, Merih Kızıl Çakar, Turhan Caglar, Fevzi Altuntas

**Affiliations:** 1 Department of Hematology and Bone Marrow Transplantation, Dr. Abdurrahman Yurtaslan Ankara Oncology Training and Research Hospital, Ankara, TUR; 2 Obstetrics, Etlik Zubeyde Hanım EAH, Ankara, TUR; 3 Obstetrics and Gynecology, Etlik Zubeyde Hanım EAH, Ankara, TUR; 4 Department of Hematology, Dr. Abdurrahman Yurtaslan Ankara Oncology Training and Research Hospital, Ankara, TUR

**Keywords:** placenta diseases, miscarriage, obstetric complications, jak2 mutation, essential thrombocythemia

## Abstract

Background

Essential thrombocythemia (ET) is a chronic myeloproliferative neoplasm characterized by persistently elevated platelet count without a clear secondary cause. Although most patients with ET are between 55 and 60 years of age, it has been estimated that 20% of women with ET are diagnosed during reproductive ages. Miscarriage is the most frequent complication of ET that has been hypothesized to be caused by microcirculatory disturbances and placental microinfarction. Furthermore, pregnant patients with ET are at increased risk of other pregnancy complications such as preterm delivery and intrauterine growth restriction.

Methods

This study was planned to evaluate pregnancy outcomes and predictors of obstetric complications in pregnant women with essential thrombocythemia (ET). The data of 21 patients with ET were analyzed retrospectively between 2016 and 2020. Age, parity, history of miscarriage, presence of Janus kinase 2 (JAK2) mutation, history of thrombotic events, treatment of thrombocytosis during pregnancy, and obstetrical outcomes including miscarriage were compared.

Results

Patients with ET had a significantly higher rate of history of two or more previous miscarriages. Miscarriage and obstetric complications in pregnant women with ET were found to be significantly higher than in the control group. Patients with ET with obstetric complications or miscarriage more frequently had a platelet count of >1000 × 10^3^/μL. Acetylsalicylic acid (ASA) prevented miscarriages, but not obstetric complications, in patients with ET.

Conclusion

ET increases miscarriage and obstetric complications in pregnancy. Treatment with ASA may reduce pregnancy losses, but not obstetric complications.

## Introduction

Essential thrombocythemia (ET) is a chronic myeloproliferative neoplasm characterized by persistently elevated platelet count without a clear secondary cause. ET is classified under chronic myeloproliferative neoplasms [1,2]. ET occurs predominantly in women with a prevalence of 38-57 per 100,000 [2]. Although most patients with ET are between 55 and 60 years of age, a significant percentage of women with ET manifest during their reproductive years [3]. It is estimated that 20% of women with ET are diagnosed during their reproductive years [4]. The diagnostic algorithm for ET is to exclude secondary causes of thrombocytosis, including iron deficiency in patients with persistent thrombocytosis. More than half of patients with ET have Janus kinase 2 V617F (JAK2V617F) mutation. Subsequent studies have identified two other mutations that are less common in ET. These are mutations in exon 10 of the thrombopoietin receptor gene (MPL) and in exon 9 of the calreticulin gene (CALR), with CALR being more common than MPL [1].

Management of pregnant women with ET presents a particular problem. Pregnancy itself is known to increase the risk of thrombosis [5,6]. Clinicians may be concerned that the risk of thrombosis in pregnancy is further increased by the presence of ET. It has been reported that approximately one in three ET pregnancies ends in abortion in the first or second trimester [7,8]. Miscarriage is the most common complication of ET, which is thought to be caused by disturbances in the microcirculation and microinfarction of the placenta [9]. In addition, pregnant patients with ET are at increased risk for other pregnancy complications such as preterm birth and intrauterine growth restriction (IUGR) [7,8], but the literature is relatively sparse and somewhat controversial regarding the predictors of pregnancy morbidity. Some have reported that the presence of JAK2V617F mutation is an unfavorable prognostic factor, but others have not [7,10].

In the present study, we aimed to investigate pregnancy outcomes and the predictors of mortality in pregnant patients with ET. Our research questions were "Are patients with ET at increased risk for complications during pregnancy? And if so, what factors are associated with pregnancy complications?"

## Materials and methods

This retrospective study was conducted in a tertiary hospital (Etlik Zübeyde Hanım Maternity Hospital) from 2016 to 2020. The hospital database was examined to identify patients with thrombocytosis, defined as a platelet count greater than 450,000/mm^3^. Patients with transient thrombocytosis or secondary thrombocytosis were excluded. Patients who had not undergone hematological examination or had not delivered at this institution were excluded from further analysis. According to the criteria of the WHO, the web database of the Ministry of Health was searched to identify patients diagnosed with ET [3]. The eligible 21 patients were enrolled in the study. During the study period, 75,962 newborns were delivered to these facilities.

Data were examined using the patients' charts and the hospital database. The following data were used for analysis: age, parity, history of miscarriage, presence of JAK2V617F mutation, records of venous thrombosis and treatment for thrombocytosis during pregnancy, and obstetric data and complications. Acetylsalicylic acid (ASA), low-molecular-weight heparin (LMWH), and interferon used to treat thrombocytosis were obtained from medical records. The database was also searched for obstetric complications and neonatal data, including miscarriage, intrauterine fetal death, preterm birth, and IUGR. Intrauterine fetal death was defined as the death of a fetus after 16 weeks of gestation. Preterm birth was defined as deliveries that occurred before 37 weeks of gestation. Preeclampsia was defined as sustained blood pressure above 140/90 mmHg associated with proteinuria or maternal signs, symptoms, or laboratory findings [11]. Previously published criteria were used to define IUGR [12].

The control group consisted of 63 pregnant women without ET who attended antenatal care during the same period.

The present study was approved by Etlik Zübeyde Hanım Ethics Committee for Non-Experimental Investigations on August 24, 2021 (approval number: 227). Statistical analysis was performed using the Statistical Package for the Social Sciences (SPSS) version 23.0 (IBM, New York, USA). Student's T-test was performed for parametric data that were normally distributed. The Mann-Whitney U test was performed for data that were not normally distributed. The Kolmogorov-Smirnov test was performed to assess normal distribution. Fisher's exact test or Pearson's chi-square test was performed for binominal and ordinal data, as appropriate. A p-value of less than 0.05 was considered significant.

## Results

During the study period, 21 patients were identified to have ET and pregnancy. This constituted 28 patients per 100,000 deliveries. The characteristics of the patients with ET and the control group are shown in Table 1. Age, pre-pregnancy body mass index (BMI), and parity were similar in patients with essential thrombocytosis and the control group. Patients with ET had a significantly higher rate of history of two or more previous miscarriages. In addition, 33% of the patients with ET versus 6% of the patients in the control group had a history of two or more miscarriages (P = 0.006). The mean duration of the disease was 1.4 years. The disease was first diagnosed during pregnancy in 33% of the patients. JAK2V617F mutation was positive in 11 patients (52.4%). Patients with ET had a higher mean platelet count than the control group in all trimesters and in the postpartum period. Two patients (9.5%) with ET had a thrombotic episode during pregnancy. There were no cases of thrombosis in the control group. Nineteen patients received therapy during pregnancy for ET. Eighteen (85.7%) patients received ASA, 14 (66.6%) patients received LMWH, and two patients were treated with pegylated interferon alpha-2a during pregnancy.

The perinatal and neonatal outcomes of the study population and the control group are shown in Table 2. Seven pregnancies in patients with ET and five pregnancies in the control group ended in miscarriage (33% and 7.9%, respectively; P = 0.004). The rates of preeclampsia, preterm delivery, and IUGR were similar in both groups (Table 3). Composite pregnancy complications were higher in the ET group than in the control group (42.8% versus 23.8%; P = 0.039). The predictors of obstetric complications and miscarriage in patients with ET are shown in Table 3. Fewer patients without obstetric complications had a platelet count of >1000 × 10^3^/μL compared with patients with miscarriage or obstetric morbidity (25% versus 76.9%; P = 0.003). JAK2V617F positivity was detected in eight (61.5%) of patients with ET with miscarriage or morbidity and in three (37.5%) patients with ET without obstetric complications (P = 0.387). Fewer patients with miscarriage received ASA than patients without miscarriage (57.1% versus 85.7%, respectively; P = 0.021). There was no difference in the history of thrombosis, presence of JAK2V617F mutation, or platelet count of >1000 × 10^3^/μL in any trimester in patients with ET with or without miscarriage (Table 4) (P > 0.05 for all comparisons).

Of note, a single mother had recurrent unilateral pleural effusion during pregnancy. The effusion is first detected at week 20. Amniocentesis, including chromosomal microarray and cytomegalovirus and toxoplasma PCR, were negative. The effusion regressed within three weeks but later accumulated again. Therapy with pegylated interferon alpha-2a was resumed when the platelet count increased above >1000 × 10^3^/μL despite treatment with ASA. Effusion was minimal two weeks after discontinuation of treatment. This was recorded as a fetal abnormality in ET (Table 2). After delivery of the newborn, thoracentesis was performed, and chylous fluid was aspirated. The fluid reaccumulated, and a chest tube was placed. Within two weeks after birth, the chylothorax completely regressed.

**Table 1:** Clinical characteristics of patients with essential thrombocytosis and the control group. ^a^Data is a given as mean ± standard deviation. ^b^Indicates statistical significance.

	Study population (n = 21)	Control group (n = 63)	p-value
Age^a^	30 ± 6.5	30.8 ± 5.9	0.587
Pre-pregnancy BMI^a^	28.8 ± 3.8	27.7 ± 2.9	0.160
Parity			0.580
0	9 (43.8%)	20 (31.7%)	
1	6 (28.6%)	18 (28.6%)	
≥2	6 (28.6%)	25 (39.7%)	
Previous miscarriage			0.006^b^
0	10 (47.7%)	43 (68.3%)	
1	4 (19%)	16 (25.4%)	
≥2	7 (33.3%)	4 (6.3%)	
Smoking	2 (9.5%)	6 (9.5%)	1
Assisted reproduction	2 (9.5%)	3 (4.8%)	0.595
Duration of disease (mean years (minimum–maximum))	1.4 (0–11)	-	-
Thrombocytosis detected during pregnancy	7 (33%)	-	-
JAK2V617F	11 (52.4%)	-	-
Mean platelet count during pregnancy			
First trimester (×10^3^/μL)	879 ± 167	291 ± 84	<0.001^b^
Second trimester (×10^3^/μL)	805 ± 33	178 ± 29	<0.001^b^
Third trimester (×10^3^/μL)	832 ± 215	260 ± 107	<0.001^b^
Postpartum (×10^3^/μL)	799 ± 239	284 ± 107	<0.001^b^
Platelet count > 1000 × 10^3^/μL any trimester	12 (42.9%)	0	<0.001^b^
History of thrombosis	1 (4.8%)	1 (1.6%)	0.440
Thrombosis during pregnancy	2 (9.5%)	0	0.06
Treatment during pregnancy			
No treatment	2 (9.5%)	47 (74.6%)	<0.001^b^
ASA	16 (76.1%)	11 (17.5%)	<0.001^b^
LMWH	13 (61.9%)	5 (7.9%)	<0.001^b^
Pegylated interferon alpha-2a	2 (9.5%)	-	-

**Table 2:** Perinatal and neonatal outcomes of the study population and control group. ^a^Excluding miscarriages. ^b^Composite obstetrical complications defined as the presence of any of the following: preterm delivery, preeclampsia, intrauterine growth restriction, and placental abruption. ^c^Indicates statistical significance.

	Study population (n = 21)	Control group (n = 63)	p-value
Miscarriage	7 (33.3%)	5 (7.9%)	0.004^c^
Gestational age at delivery weeks (mean ± standard deviation)	38.3 ± 2.8	37.7 ± 1.2	0.146
Preterm delivery^a^	3 (21.4%)	5 (8.6%)	0.134
Preeclampsia^a^	1 (7.1%)	3 (5.2%)	0.716
Intrauterine growth restriction^a^	2 (14.3%)	3 (5.2%)	0.191
Placental abruption^a^	1 (7.1%)	1 (1.7%)	0.353
Cesarean delivery^a^	8 (57.1%)	31 (53.4%)	0.803
Primary cesarean delivery^a^	4 (28.6%)	15 (25.9%)	0.836
Composite obstetrical complications^a,b^	6 (42.8%)	10 (17.2%)	0.039^c^
Neonatal birth weight (±standard deviation)	3071 ± 642	3116 ± 418	0.747
Low-birth-weight infant	2 (15.4%)	6 (10.3%)	0.603
Fetal anomaly	1 (7.1%)	1 (1.7%)	0.353

**Table 3:** Predictors of obstetric complications in patients with essential thrombocytosis. ^a^Composite obstetric morbidity defined as the presence of any of the following: preterm delivery, preeclampsia, intrauterine growth restriction, and placental abruption. ^b^Indicates statistical significance.

	Patients with miscarriage or obstetric morbidity^a^ (n = 13)	Patients without complications (n = 8)	p-value
Treatment type			
No treatment	2 (15.3%)	0	0.505
ASA treatment	8 (61.5%)	8 (100%)	0.110
LMWH	7 (53.8%)	7 (87.5%)	0.173
Pegylated interferon alpha-2a treatment	0	2 (25%)	0.467
Platelet count > 1000 × 10^3^/μL any trimester	10 (76.9%)	2 (25%)	0.003^b^
History of thrombosis	0	1 (12.5%)	0.381
Presence of JAK2V617F	8 (61.5%)	3 (37.5%)	0.387

**Table 4:** Factors associated with miscarriage in patients with essential thrombocytosis. ^a^Indicates statistical significance.

	Patients with miscarriage (n = 7)	Patients without miscarriage (n = 14)	p-value
Treatment type			
No treatment	2 (28.6%)	0	0.100
ASA treatment	4 (57.1%)	13 (92.9%)	0.021^a^
LMWH	4 (57.1%)	12 (85.7%)	0.119
Pegylated interferon alpha-2a treatment	0	2 (14.2%)	0.533
Platelet count > 1000 × 10^3^/μL any trimester	5 (71.4%)	7 (50%)	0.642
History of thrombosis	0	2 (14.2%)	0.533
Presence of JAK2V617F mutation	4 (57.1%)	7 (50%)	0.725

## Discussion

In this study, we examined the perinatal and neonatal outcomes of pregnant women with ET. In our study, a history of two or more miscarriages was significantly higher in pregnant women with ET than in the control group. JAK2V617F mutation was found positive in 52.4% of the pregnant women with ET, and miscarriages occurred significantly more frequently in patients with ET than in the control group. Composite obstetric complications were significantly higher in pregnant women with ET than in the control group. A platelet count of >1000 × 10^3^/μL in any trimester in pregnancy was associated with pregnancy loss or obstetric morbidity. Patients whose pregnancies continued beyond the first trimester were more likely to receive ASA during pregnancy. The rate of JAK2V617F mutation was not different in pregnant women with and without complications and miscarriage (Table 3).

Polycythemia vera (PV), essential thrombocythemia (ET), and primary myelofibrosis (PMF) belong to the category of Philadelphia negative, the so-called classical MPNs. The potential applications of liquid biopsy and liquid biopsy-based biomarkers have not yet been explored in MPNs [13]. Although cfDNA has been detected primarily by liquid biopsy in solid cancers and various lymphoma subtypes, a few studies have examined cfDNA in MPNs. However, in this small number of studies, it was hypothesized that cfDNA could be used to distinguish MPNs from healthy individuals and between MPN subtypes, i.e., to distinguish PMF from PV and ET [13].

The fetal loss rate increases in the first trimester in patients with ET. Indeed, miscarriage is a common complication in pregnant women with ET. In our study, pregnancy loss was observed in one-third of the patients. Melillo et al. in their study examining the outcome of 122 pregnant women with ET reported that the rate of pregnancies resulting in miscarriage was 21.3% [14]. It was reported that the presence of JAK2V617F mutation and treatment with pegylated interferon alpha-2a reduced the risk of miscarriage, but treatment with ASA did not [14]. Gangat et al. reported a miscarriage rate of 35% in 36 pregnant women with ET [7]. In this study, they investigated the risk factors for first trimester losses. It was reported that the use of ASA in the first trimester significantly reduced the risk of miscarriage. However, in this study, miscarriage was not associated with JAK2V617F mutation or high platelet count [7]. Passamonti et al. found an association between JAK2V617F mutation and pregnancy loss, but they did not find an association between treatment with ASA and pregnancy loss [8]. Our results are consistent with those of Gangat et al. [7], but not with those of Passamonti et al. and Melillo et al. [8,14]. Kwiatkowski et al. reported in their study, in which they followed 52 pregnancies of 27 patients with ET, that the live birth rate increased with ASA and LMWH [10]. In contrast, JAK2V617F mutation, pegylated interferon alpha-2a treatment, or platelet count were not associated with miscarriage risk. We agree with these results because ASA was used less frequently in patients who miscarried. As mentioned earlier, previous studies reach conflicting conclusions regarding risk factors for miscarriage and the benefits of treatment from ET. Because of the retrospective nature of all studies, confounding factors for miscarriage, such as fetal chromosomal abnormalities, have not been identified. In addition, it may not be known exactly at which gestational week ET treatment is started, which may have led to inconsistent results regarding the efficacy of the therapy in preventing miscarriage. In our study, ET patients who suffered miscarriage were treated with ASA to a lesser extent than those whose pregnancies continued. However, there were no other factors associated with miscarriage in patients with ET.

Many studies have examined the live birth rate in patients treated with ET. However, the literature has reported that ET has been associated with pregnancy complications other than miscarriage. Kaibara et al. and Mercer et al. reported cases of intrauterine death with placental infarction in pregnant women with ET [15,16]. Falconer et al. reported fetal growth retardation in pregnant women with ET with placental infarcts [17]. In our study, the rate of preeclampsia, preterm delivery, and IUGR was similar in both groups. However, when obstetric complications were evaluated cumulatively, the cumulative risk of obstetric complications was significantly increased in the patients with ET compared with the control group (Table 3). One of the crucial findings of our study is that pregnancy complications, including miscarriage, increased in patients with a high platelet count (>1000 × 10^3^/μL). Previous studies have not found an association between platelet count and pregnancy complications [8,10]. However, the focus of these studies was on live birth rate, and pregnancy complications were not investigated. A recent study reported that miscarriages and other pregnancy complications increased in pregnant women with ET. However, there was no control group in this study [18].

The treatment of ET in pregnancy is antiplatelet therapy, especially ASA. ASA has been used in all previous studies, although controversial results have been observed regarding its effects on miscarriage [7,8]. In our study, the rate of treatment with ASA was similar in patients with ET with obstetrical complications, including miscarriage, and patients with ET without obstetrical complications. A recent study reported that the use of LMWH and acetylsalicylic acid in patients with ET increased the live birth rate to 100% [10]. We did not find LMWH beneficial in preventing miscarriage or obstetrical complications. However, LMWH was frequently used concomitantly with ASA in the present study, which might shadow the potential therapeutic benefit of LMWH. We believe that ASA will continue to be the main component of treatment for these pregnant women because it is safe in pregnancy. It has been shown in previous studies to prevent some of the morbidities such as preeclampsia. However, due to the ASA resistance seen in some patients, which was stated in the ASPRE study, treatment with higher doses of ASA in patients with ET and its use in early pregnancy should be investigated [19].

The present study has some limitations. It is not a prospective study, and it is limited to data from a single center. In addition, the number of patients is relatively small. However, it is one of the few studies that investigated obstetric complications other than miscarriage.

## Conclusions

In conclusion, ET is associated with an increased number of pregnancy complications and miscarriages. Pregnancy complications are more common in patients with high platelet counts. Treatment with ASA may reduce pregnancy losses, but it is uncertain whether antiplatelet therapy can reduce obstetrical complications in these patients. Prospective studies are needed to clarify the timing of treatment (e.g., early first trimester), ASA dose and combination therapy, and optimal platelet count in the periconceptional period.

## References

[1] Robinson AJ, Godfrey AL (2021). Low-risk essential thrombocythemia: a comprehensive review. Hemasphere.

[2] Arber DA, Orazi A, Hasserjian R (2016). The 2016 revision to the World Health Organization classification of myeloid neoplasms and acute leukemia. Blood.

[3] Mehta J, Wang H, Iqbal SU, Mesa R (2014). Epidemiology of myeloproliferative neoplasms in the United States. Leuk Lymphoma.

[4] Burbury K, Panigrahi A (2021). Esssential thrombocythaemia and pregnancy-a need for prospective study and a consensus on its management. Leuk Res.

[5] McNally RJ, Roman E, Cartwright RA (1999). Leukemias and lymphomas: time trends in the UK, 1984-93. Cancer Causes Control.

[6] Pomp ER, Lenselink AM, Rosendaal FR, Doggen CJ (2008). Pregnancy, the postpartum period and prothrombotic defects: risk of venous thrombosis in the MEGA study. J Thromb Haemost.

[7] Gangat N, Wolanskyj AP, Schwager S, Tefferi A (2009). Predictors of pregnancy outcome in essential thrombocythemia: a single institution study of 63 pregnancies. Eur J Haematol.

[8] Passamonti F, Randi ML, Rumi E (2007). Increased risk of pregnancy complications in patients with essential thrombocythemia carrying the JAK2 (617V>F) mutation. Blood.

[9] Yu Y, Zhang X, Shi Q, Wang M, Jing J, Liu Y (2019). Essential thrombocytosis with recurrent spontaneous abortion in the mid trimester: a case report. Medicine (Baltimore).

[10] Kwiatkowski J, Kuliszkiewicz-Janus M, Potoczek S, Jaźwiec B, Wróbel T, Małecki R (2021). What factors determine the pregnancy outcome in patients with essential thrombocythemia?. J Matern Fetal Neonatal Med.

[11] (2013). Hypertension in pregnancy. Report of the American College of Obstetricians and Gynecologists’ Task Force on Hypertension in Pregnancy. Obstet Gynecol.

[12] Gordijn SJ, Beune IM, Thilaganathan B (2016). Consensus definition of fetal growth restriction: a Delphi procedure. Ultrasound Obstet Gynecol.

[13] Găman MA, Cozma MA, Dobrică EC, Crețoiu SM, Găman AM, Diaconu CC (2021). Liquid biopsy and potential liquid biopsy-based biomarkers in Philadelphia-negative classical myeloproliferative neoplasms: a systematic review. Life (Basel).

[14] Melillo L, Tieghi A, Candoni A (2009). Outcome of 122 pregnancies in essential thrombocythemia patients: a report from the Italian registry. Am J Hematol.

[15] Kaibara M, Kobayashi T, Matsumoto S (1985). Idiopathic thrombocythemia and pregnancy: report of a case. Obstet Gynecol.

[16] Mercer B, Drouin J, Jolly E, d'Anjou G (1988). Primary thrombocythemia in pregnancy: a report of two cases. Am J Obstet Gynecol.

[17] Falconer J, Pineo G, Blahey W, Bowen T, Docksteader B, Jadusingh I (1987). Essential thrombocythemia associated with recurrent abortions and fetal growth retardation. Am J Hematol.

[18] How J, Leiva O, Bogue T (2020). Pregnancy outcomes, risk factors, and cell count trends in pregnant women with essential thrombocythemia. Leuk Res.

[19] Rolnik DL, Wright D, Poon LC (2017). ASPRE trial: performance of screening for preterm pre-eclampsia. Ultrasound Obstet Gynecol.

